# *Trianthema portulacastrum* Linn. Displays Anti-Inflammatory Responses during Chemically Induced Rat Mammary Tumorigenesis through Simultaneous and Differential Regulation of NF-κB and Nrf2 Signaling Pathways

**DOI:** 10.3390/ijms16022426

**Published:** 2015-01-22

**Authors:** Animesh Mandal, Anupam Bishayee

**Affiliations:** 1 Cancer Therapeutics and Chemoprevention Group, Department of Pharmaceutical Sciences, College of Pharmacy, Northeast Ohio Medical University, Rootstown, OH 44272, USA; E-Mail: animandal0@gmail.com; 2 Department of Pharmaceutical and Biomedical Sciences, College of Pharmacy, California Northstate University, Elk Grove, CA 95757, USA

**Keywords:** breast tumor, DMBA, *Trianthema portulacastrum*, COX-2, HSP90, NF-κB, Nrf2, anti-inflammatory mechanisms

## Abstract

*Trianthema portulacastrum*, a medicinal and dietary plant, has gained substantial importance due to its various pharmacological properties, including anti-inflammatory and anticarcinogenic activities. We have recently reported that a characterized *T. pofrtulacastrum* extract (TPE) affords a considerable chemoprevention of 7,12-dimethylbenz(a)anthracene (DMBA)-induced rat mammary tumorigenesis though the underlying mechanisms are not completely understood. The objective of this study was to investigate anti-inflammatory mechanisms of TPE during DMBA mammary carcinogenesis in rats by monitoring cyclooxygenase-2 (COX-2), heat shock protein 90 (HSP90), nuclear factor-kappaB (NF-κB) and nuclear factor erythroid 2-related factor 2 (Nrf2). Mammary tumors were harvested from our previous study in which TPE (50–200 mg/kg) was found to inhibit mammary tumorigenesis in a dose-response manner. The expressions of intratumor COX-2, HSP90, NF-κB, inhibitory kappaB-alpha (IκBα) and Nrf2 were determined by immunohistochemistry. TPE downregulated the expression of COX-2 and HSP90, blocked the degradation of IκBα, hampered the translocation of NF-κB from cytosol to nucleus and upregulated the expression and nuclear translocation of Nrf2 during DMBA mammary carcinogenesis. These results in conjunction with our previous findings suggest that TPE prevents DMBA-induced breast neoplasia by anti-inflammatory mechanisms mediated through simultaneous and differential modulation of two interconnected molecular circuits, namely NF-κB and Nrf2 signaling pathways.

## 1. Introduction

*Trianthema portulacastrum* Linn. (family: Aizoaceae) is an exotic plant of Southeast Asia, tropical America and Africa [1]. The plant is capable of growing in sunny desert areas, including Arizona, and also grows abundantly as a “weed” in well irrigated and high rainfall areas, particularly in India and neighboring countries of Bangladesh, Pakistan and Sri Lanka. *T. portulacastrum* is used as a valuable herb in the Indian traditional medicinal system, such as Ayurvedic medicine [1]. In India and other South-East Asian countries, *T. portulacastrum* is commonly used in vegetable dishes during the rainy seasons when it grows abundantly. In Africa, especially Ghana and Tanzania, the young leaves of the plant are consumed as cooked vegetables or in soups [2]. Recent study showed nutritional potential of this wild edible plant as it represents a good source of fiber, proteins, riboflavin, potassium, sodium and iron [3].

Several anatomical parts of *T. portulacastrum* are traditionally used as analgesic, alexiteric, alterative, laxative and stomachic and also valuable for the treatment of alcohol poisoning, anemia, ascites, asthma, beri-beri, bronchitis, corneal ulcers, dropsy, edema, heart diseases, inflammation, liver ailments, migraine, night blindness, piles and rheumatism [1,4,5,6]. Based on scientific investigation, various extracts of and isolated phytochemicals from *T. portulacastrum* have been found to possess a number of pharmacological properties, including analgesic, antibacterial, antifungal, anti-inflammatory, antioxidant, antipyretic, hypoglycemic and hypolipidemic activities [7,8,9,10,11].

Our laboratory has previously reported that an ethanolic extract of the whole plant of *T. portulacastrum* (excluding the roots) exerted a potent hepatoprotective activity against alcohol-carbon tetrachloride (CCl_4_)-induced acute [12] and chronic liver damage in mice [13]. Supplementary studies have confirmed the hepatoprotective activity of *T. portulacastrum* which has been caused through regulation of erythropoiesis and general immunity [14], activities of hepatic antioxidant defense enzymes [15], and hepatic oxidative DNA damage and chromosomal aberrations [16] in CCl_4_-intoxicated mice. Consequently, an ethanolic leaf extract of *T. portulacastrum* has been found to exhibit antihepatotoxic effects against hepatic damage inflicted by paracetamol and thioacetamide [17] as well as aflatoxin B1 [18,19] in rats. Another study has demonstrated a protective effect of a methanolic extract of the whole plant of *T. portulacastrum* against atherosclerotic diet-induced hepatic and renal disorders in rats [20].

A significant hepatoprotective activity *T. portulacastrum* has stimulated interest in exploring antihepatocarcinogenic potential of this dietary and medicinal plant. Various extracts (aqueous, ethanolic and chloroform) prepared using overground parts of *T. portulacastrum* have been found to lower the incidence, multiplicity and size of visible neoplastic hepatic nodules as well as microscopic altered liver cell foci induced by diethylnitrosamine (DENA), a potent hepatocarcinogen, in rats [21]. A follow-up study has demonstrated that the aforementioned extracts modulated hepatic enzyme activities of phase I and II drug metabolism and antioxidant defense in DENA-treated rats [22]. The chloroform extract has also been found to exhibit an inhibitory effect against rat hepatocellular carcinogenesis initiated with DENA and promoted by phenobarbital [23].

Numerous natural products, including phytochemicals, have been shown to kill mammary tumor cells and prevent the occurrence or suppress the growth of breast tumors in animal models by modulation of proliferation, differentiation, apoptosis, oxidative stress, inflammation, angiogenesis and various cell signaling pathways [24,25,26]. We have initiated a comprehensive research program to explore chemopreventive effect of *T. portulacastrum* against breast cancer. Recently, we have provided compelling experimental evidence for the first time that dietary administration of a characterized ethanolic extract of *T. portulacastrum* exhibits a striking suppression of 7,12-dimethyl benz(*a*)anthracene (DMBA)-initiated mammary tumor incidence, total tumor burden and average tumor weight in female Sprague-Dawley rats without any toxic manifestation [27]. Mechanistic study has revealed that *T. portulacastrum* extract (TPE) dose-dependently inhibits abnormal cellular proliferation, induces apoptosis, upregulates proapoptotic protein Bax, downregulates antiapoptotic protein Bcl-2 and diminishes activated Wnt/β-catenin signaling in rat mammary tumors induced by DMBA [27]. In addition to these encouraging results, other distinct or complimentary mechanisms could be involved in mammary tumor-inhibitory effect of *T. portulacastrum*. It is well established that chronic inflammatory conditions are involved in the development and progression of mammary carcinoma [28,29,30,31,32] and *T. portulacastum* constituents possess anti-inflammatory properties reviewed in [11]. Accordingly, we have hypothesized that TPE exerts inhibition of DMBA-induced mammary tumorigenesis at least, in part, by suppression of inflammatory stress response. Thus, our current study aims to investigate anti-inflammatory mechanisms of TPE by monitoring several proinflammatory and stress markers, namely cyclooxygenase-2 (COX-2) and heat shock protein 90 (HSP90), and various inflammation-regulatory signaling pathways, namely nuclear factor-κB (NF-κB) and nuclear factor erythroid 2-related factor 2 (Nrf2), during DMBA-evoked mammary gland neoplasia in rats.

## 2. Results

### 2.1. TPE Suppresses Elevated COX-2 Expression during DMBA-Induced Mammary Tumorigenesis

**Figure 1:**
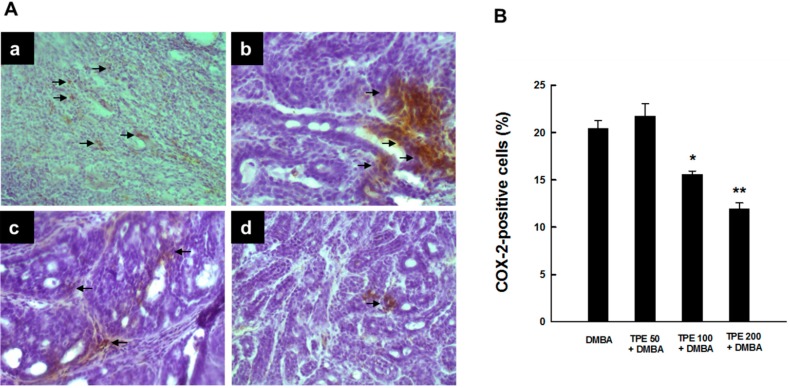
Effect of TPE on COX-2 expression in DMBA-induced breast tumors in female Sprague-Dawley rats. The rats had free access to food with or without TPE two weeks prior to, and 16 weeks following, DMBA administration. All animals were sacrificed 18 weeks following the commencement of the study, tumor tissues were harvested and used for the assay using anti-COX-2 antibody. (**A**) Immunohistochemical localization of COX-2-positive cells in tumor sections. Arrows indicate immunohistochemical staining of COX-2 (magnification: ×200). (**a**) Intense COX‑2 immunoreactivity in DMBA control; (**b**) minimal increase in COX‑2 expression in TPE (50 mg/kg) plus DMBA group; (**c**) extensive decrease in COX‑2 expression in TPE (100 mg/kg) plus DMBA group; and (**d**) very limited expression of COX-2 in TPE (200 mg/kg) plus DMBA group; (**B**) Quantitative analysis of COX-2-immunopositive cells during DMBA mammary tumorigenesis in rats in the presence or absence of TPE treatment. Results are based on 1000 cells per animal and 4 animals per group. Each bar represents the mean ± SEM (*n* = 4). * *p* < 0.01 and ** *p* < 0.001 compared to DMBA control.

Since chronic inflammation plays a crucial role in mammary carcinogenesis [28,29,30,31,32], we have investigated the ability of TPE to inhibit expression of the inflammatory enzyme COX-2. Immunostaining of COX-2 protein in mammary tumor specimens obtained from rats subjected to DMBA treatment in the presence or absence of TPE administration revealed a considerable expression of COX-2 predominantly in the cytoplasm of tumor cells from DMBA control animals (Figure 1A-a). TPE at a dose of 50 mg/kg did not influence the magnitude of intratumor COX-2 immunopositivity compared to DMBA control (Figure 1A-b). However, a substantial and drastic suppression of COX-2 expression was noticed in DMBA-induced mammary tumors from rats treated with TPE at a dose of 100 and 200 mg/kg, respectively (Figure 1A-c and Figure 1A-d). Figure 1B depicts the percentage of COX-2-positive cells in tumor sections from all experimental animal groups. Our quantitative analysis shows immunopositivity for more than 20% of mammary tumor cells in DMBA control animals. TPE at 50 mg/kg slightly increased the percentage of COX-2-positive cells; however the result did not reach the level of statistical significance. Interestingly, there was a significant (*p* < 0.01 or 0.001) inhibition of the percentage of COX-2-positive tumor cells in rats fed with 100 or 200 mg/kg TPE compared to the DMBA control group, respectively.

### 2.2. TPE Inhibits HSP90 Expression during DMBA Mammary Carcinogenesis

**Figure 2:**
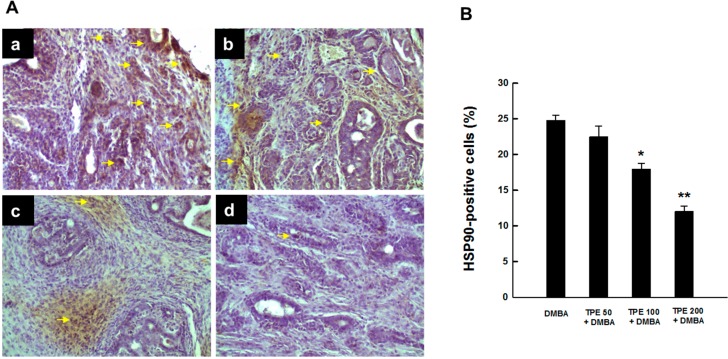
Expression of HSP90 during DMBA-initiated mammary gland tumorigenesis in rats in the presence or absence of TPE treatment. The animals were treated as indicated in legend to Figure 1. The mammary tumor sections were subjected to immunohistochemical analysis using anti-HSP90 antibody. (**A**) Immunohistochemical localization of HSP90-positive cells in tumor samples. Arrows indicate immunohistochemical staining of HSP90 (magnification: ×200). Various treatment groups are: (**a**) DMBA control; (**b**) TPE (50 mg/kg) plus DMBA; (**c**) TPE (100 mg/kg) plus DMBA; and (**d**) TPE (200 mg/kg) plus DMBA; (**B**) Quantitative analysis of HSP90-positive cells during DMBA mammary tumorigenesis in rats in the presence or absence of TPE treatment. Results are based on 1000 cells per animal and four animals per group. Each bar represents the mean ± SEM (*n* = 4). * *p* < 0.01 and ** *p* < 0.001 compared to DMBA control.

Since HSP90 is induced in breast cancer [33,34], we determined the protein expression of this molecular chaperone in DMBA-induced mammary tumors in rats in the presence or absence of TPE treatment using immunohistochemical techniques. An extensive expression of intratumor HSP90 was found in DMBA control animals (Figure 2A-a). Somewhat similar results were observed in rats subjected to TPE treatment at 50 mg/kg in conjunction with DMBA carcinogenesis (Figure 2A-b). The expression of HSP90 was prominently reduced in tumor samples obtained from animals treated with TPE at 100 or 200 mg/kg compared to DMBA control (Figure 2A-c and Figure 2A-d). The quantitative analyses of HSP90-immunopositive cells revealed a dose-dependent suppression of this protein expression in tumor sections from all DMBA-treated rats that received TPE treatment (Figure 2B). Nevertheless, the results were statistically significant (*p* < 0.01 or 0.001) in the group treated with 100 or 200 mg/kg TPE, respectively.

### 2.3. TPE Attenuates Activation of NF-κB during Mammary Tumorigenesis

**Figure 3:**
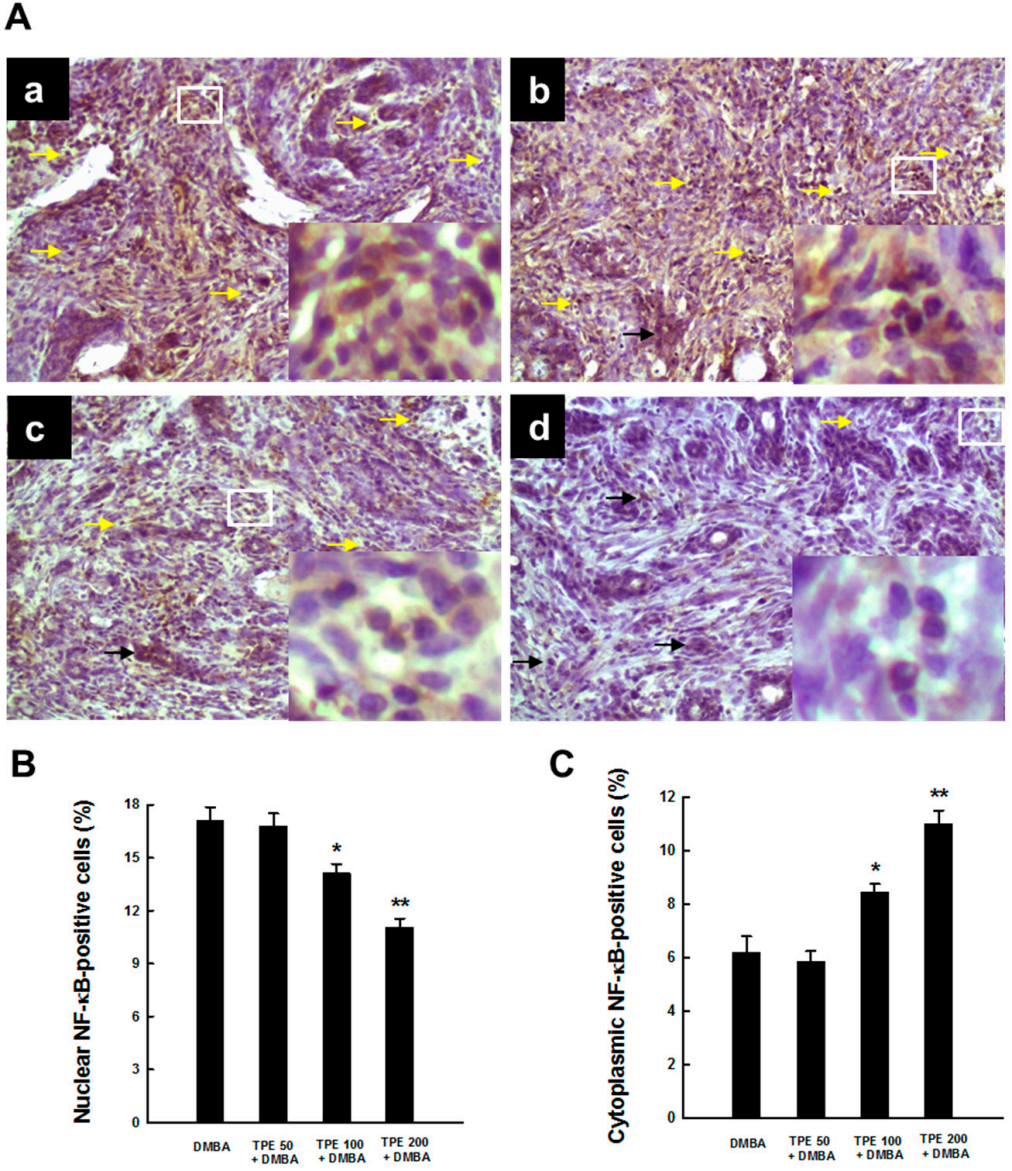
Effect of TPE on NF-κB p65 activation during DMBA-induced mammary gland carcinogenesis in female Sprague-Dawley rats. The animals were treated as indicated in the legend to Figure 1. The mammary tumor sections were subjected to immunohistochemical analysis using anti-NF-κB p65 antibody. (**A**) Representative immunohistochemical localization of NF-κB p65 in nucleus (yellow arrows) and cytoplasm (black arrows) are depicted (magnification: × 200). Various treatment groups are: (**a**) DMBA control; (**b**) TPE (50 mg/kg) plus DMBA; (**c**) TPE (100 mg/kg) plus DMBA; and (**d**) TPE (200 mg/kg) plus DMBA. The nuclear expression of NF-κB p65 in the designated area marked by the white box is shown as an inset (magnification: ×1000) for each treatment group. Quantitative analysis of (**B**) nuclear and (**C**) cytoplasmic NF-κB-immunopositive cells in rat mammary tumors induced by DMBA in the presence or absence of TPE treatment. Results are based on 1000 cells per animal and 4 animals per group. Each bar represents the mean ± SEM (*n* = 4). (**B**) * *p* < 0.05 and ** *p* < 0.001 compared to DMBA control. (**C**) * *p* < 0.01 and ** *p* < 0.001 compared to DMBA control.

NF-κB, a transcription factor, represents a cardinal regulator of inflammation and persistent (constitutive) activation of NF-κB contributes to the development and progression of a number of cancers, including breast cancer [29,35,36]. Hence, we assessed the activation of NF-κB in mammary tumor sections harvested from rats exposed to DMBA in the presence or absence of TPE treatment. Our immunohistochemical results demonstrate a considerable expression of NF-κB p65 in nucleus and very limited expression of the same protein in cytoplasm of tumor sections obtained from DMBA control animals, indicating activation and subsequent translocation of NF-κB p65 from cytosol to nucleus (Figure 3A-a). Nearly similar expression patterns of nuclear and cytoplasmic NF-κB p65 were observed following the treatment with TPE at 50 mg/kg (Figure 3A-b). On the other hand, TPE at 100 or 200 mg/kg decreased nuclear NF-κB expression and increased the expression of the same protein in the cytosol (Figure 3A-c and Figure 3A-d, respectively). The quantitative analysis of NF-κB p65-immuno-positive cells shows a significant (*p* < 0.05 or 0.001) decrement in nuclear NF-κB p65-positive cells (Figure 3B) and a significant (*p* < 0.01 or 0.001) increment in cytoplasmic NF-κB p65-positive cells (Figure 3C) in two TPE-treated groups (100 and 200 mg/kg) compared to DMBA control.

**Figure 4:**
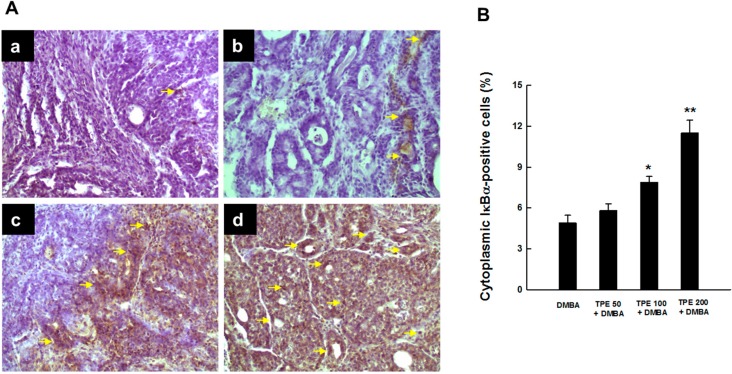
The immunohistochemical expression of IκBα during DMBA-evoked mammary neoplasia in rats in the presence or absence of TPE. The animals were treated as indicated in the legend to Figure 1. The mammary tumor sections were subjected to immunohistochemical analysis using anti-IκBα antibody. (**A**) Immunohistochemical localization of IκBα-positive cells in tumor samples. Arrows indicate immunohistochemical staining of IκBα in cytoplasm (magnification: ×200). Various treatment groups are: (**a**) DMBA control; (**b**) TPE (50 mg/kg) plus DMBA; (**c**) TPE (100 mg/kg) plus DMBA; and (**d**) TPE (200 mg/kg) plus DMBA; (**B**) Quantification of cytoplasmic IκBα-immunopositive cells in rat mammary tumors induced by DMBA in the presence or absence of TPE treatment. Results are based on 1000 cells per animal and four animals per group. Each bar represents the mean ± SEM (*n* = 4). * *p* < 0.05 and ** *p* < 0.001 compared to DMBA control.

Since degradation of inhibitory kappaB-alpha (IκBα) embodies an essential step for the activation of NF-κB [37], we examined whether suppression of DMBA-induced activation of NF-κB by TPE was due to the inhibition of IκBα degradation. As a matter of fact, we noticed very limited expression of cytosolic IκBα in DMBA control animals (Figure 4A-a), indicating possible degradation of IκBα protein. Oral TPE treatment reversed DMBA-induced degradation of IκBα protein in cytosol in a dose-dependent manner. Our results also revealed a modest increase in cytosolic IκBα expression by 50 mg/kg TPE (Figure 4A-b) and substantial upregulation of the same protein by 100 or 200 mg/kg TPE (Figure 4A-c and Figure 4A-d, respectively). These qualitative results are supported by quantitative analysis of IκBα-positive cells that displayed a significant (*p* < 0.05 or 0.001) increment of immunopositive cells in the mammary tumor sections obtained from rats treated with 100 or 200 mg/kg TPE (Figure 4B).

### 2.4. TPE Upregulates Nrf2 Expression during Mammary Tumorigenesis Induced by DMBA

**Figure 5:**
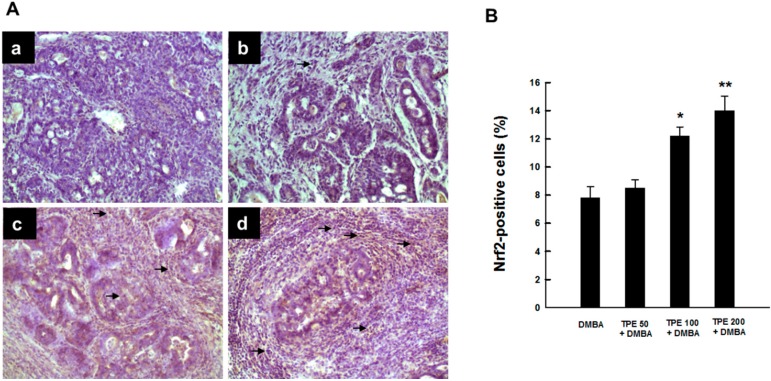
Effect of TPE on intratumor Nrf2 expression during DMBA-initiated breast carcinogenesis in rats. The animals were treated as indicated in legend to Figure 1. The mammary tumor sections were subjected to immunohistochemical analysis using anti-Nrf2 antibody. (**A**) Representative immunohistochemical localization of Nrf2 in nucleus. Arrows indicate immunohistochemical staining of Nrf2 (magnification: ×200). Various treatment groups are: (**a**) DMBA control; (**b**) TPE (50 mg/kg) plus DMBA; (**c**) TPE (100 mg/kg) plus DMBA; and (**d**) TPE (200 mg/kg) plus DMBA; (**B**) Quantification of nuclear Nrf2-immunopositive cells in rat mammary tumors induced by DMBA in the presence or absence of TPE treatment. Results are based on 1000 cells per animal and four animals per group. Each bar represents the mean ± SEM (*n* = 4). * *p* < 0.01 and ** *p* < 0.001 compared to DMBA control.

Since Nrf2 is highly relevant to inflammation-driven carcinogenesis and there exists a possible crosstalk between Nrf2 and NF-κB signaling pathways [38,39], we investigated the involvement of Nrf2 in DMBA-induced mammary carcinogenesis and its possible modulation by TPE. Figure 5A shows the immunohistochemical profiles of Nrf2 expression in tumor sections originated from various rat groups. The DMBA control group showed very limited expression of intratumor Nrf2 (Figure 5A-a). TPE treatment at 50 mg/kg marginally increased the expression of Nrf2 (Figure 5A-b). In contrast, tumor sections from DMBA-initiated animals treated with 100 or 200 mg/kg TPE showed a drastic upregulation in the expression of Nrf2-positive cells (Figure 5A-c and Figure 5A-d). The preponderance of Nrf2-immono-positivity was noticed in the nucleus, indicating activation of this nuclear factor and its subsequent translocation from the cytoplasm to the nucleus. Quantitative analysis revealed an increase in the percentage of Nrf2-positive cells due to TPE treatment in a dose-responsive fashion compared to DMBA control. However, a statistically significant (*p* < 0.01 or 0.001) result was obtained with 100 or 200 mg/kg TPE, respectively.

## 3. Discussion

Breast cancer is the most common cancer among women and one of the most leading causes of death in women worldwide. In the United States, breast cancer represents the second most common female cancer following skin cancer. According to the American Cancer Society, approximately 232,700 new cases of breast cancer are estimated to occur in women in the United States in 2014 [40]. Breast cancer is also the second most important cause of cancer-related death in American women with 40,000 predicted deaths in 2014 [40]. Empirical evidence suggests the role of inflammation in breast cancer pathogenesis and progression [28,29,30]. Several inflammatory signaling pathways are constitutively activated in various types of breast cancer, including inflammatory breast cancer (IBC). Various inflammatory molecules and signaling pathways are implicated in malignant transformation, proliferation, survival, epithelial-mesenchymal transition, invasion and metastasis of breast cancer cells [41]. Hence, inhibitors of inflammation may be valuable in the prevention and treatment of breast cancer. Several pharmacological compounds, natural constituents and dietary agents endowed with anti-inflammatory mechanisms have shown promise in the prevention and intervention of mammary cancer [42,43,44,45,46]. Recently, we have reported the novel finding that a characterized extract from Indian medicinal and dietary plant *T. portulacastrum* affords a striking chemoprevention of DMBA-induced rat mammary tumorigenesis though the underlying mechanism of action of such effect is not completely understood [27]. Since several phytochemicals present in *T. portulacastum* are known to exhibit anti-inflammatory properties [11], TPE-mediated inhibition of DMBA mammary carcinogenesis could involve interference with the inflammatory cascade. Hence, the current work was designed as an extension of our previous study to investigate the ability of TPE to impede DMBA-evoked inflammatory signaling pathways by utilizing breast tumor samples harvested from our previously completed chemopreventive study [27].

Numerous studies have suggested a connection between the development of breast cancer and the prostaglandin (PG) synthesis pathway regulated by the COX family of PG synthases [47,48]. While COX-1 is constitutively expressed in tissues, the expression of COX-2 is induced by various stimuli, such as shear stress, cytokines, growth factors and oncogenes [49]. Both enzymes are involved in the conversion of arachidonic acid to prostanoids and are also responsible for the production of eicosanoids, which trigger pain and inflammation. PGs produced by COX-2 are involved in various critical steps in malignancy, such as cell proliferation, mutagenesis, apoptosis evasion, immune suppression, angiogenesis and invasion [50]. Although COX-2 remains mostly undetectable in normal breast tissue by immunohistochemistry, elevated COX-2 enzyme levels have been found in approximately 40% of human breast carcinoma samples [47]. A recent meta-analysis showing similar COX-2 protein expression in ductal carcinoma *in situ* as well as invasive breast cancer indicates the critical role of COX-2 in early stages of mammary carcinogenesis [51]. All these findings strongly support the involvement of COX-2 in mammary neoplasia and the rationale of using the COX-2 pathway as a target for breast cancer prevention and treatment. In our present study, analysis of COX-2 protein expression by immunohistochemistry revealed substantial expression of COX-2 in DMBA-initiated mammary tumors, supporting observations made by other laboratories [52,53,54]. Our results, for the first time, demonstrate TPE-mediated suppression of COX-2 in experimentally induced mammary carcinogenesis in rats. We propose that our previously observed mammary tumor suppressive effect of TPE in during DMBA carcinogenesis [27] could be, in part, due to inhibition of COX-2 expression and thereby suppression of PG synthesis. Our results with TPE are in agreement with various dietary agents and natural products showing chemopreventive efficacy in several chemical mammary carcinogenesis models by simultaneous downregulation of COX-2 protein [53,54,55,56].

HSPs, a family of stress-inducible proteins, are known to play vital roles in the cellular stress response. Experimental evidence suggests that HSP90 may regulate inflammatory events through modulation of cytokines and various cell signaling pathways [57,58,59]. An activated multi-chaperone complex of HSP90 has been detected in tumor cells [60] and elevated expressions of HSP90 have been associated with poor prognosis in patients with mammary carcinoma [33,34]. The interaction of HSP90 with various oncogenic pathways makes this protein a viable target for cancer therapy and a number of clinical trials are evaluating the potential of various HSP90 inhibitors for the treatment of several neoplastic diseases, including breast cancer [61,62,63]. We have used immunohistochemical technique to analyze the HSP90 protein expression during DMBA-induced rat mammary carcinogenesis in the presence or absence of TPE treatment. A high level of intratumor HSP90 expression in DMBA group indicates that DMBA may exert some degree of heat shock response possibly due to inflammatory stress within mammary tumor cells resulting in irregular proliferation and evasion of apoptosis as we reported previously [24]. Our results showing significantly reduced expression of HSP90 in the TPE plus DMBA group suggest the ability of TPE to depress mammary tumor cell growth and survival by downregulation of HSP90 expression, which may be viewed as an indication of reduced inflammatory stress. This study also provides preclinical evidence of achieving breast cancer chemoprevention by targeting HSP90. In line with our present finding, several natural substances have been shown to suppress experimental tumorigenesis, including rat mammary carcinogenesis, through downregulation of HSP90 [64,65,66].

Numerous signaling pathways implicated in tumorigenesis are likely to be networked through the activation of proinflammatory transcription factor NF-κB [67]. The major inactive form of NF-κB complex, a p50-p65 heterodimer that binds to inhibitory protein IκBα, resides primarily in the cytoplasm. In the canonical (classical) pathway triggered by proinflammatory cytokines, including tumor necrosis factor-α and interleukin-1β, IκBα is subjected to degradation by the NF-κB essential modulator/IκB kinase (IKK)γ-containing IKK complex via a transforming growth factor-β-activated kinase 1-dependent pathway [68]. Consequently, the released p50-p65 dimer translocates into the nucleus, binds to its cognate response element in the DNA, and induces the transcription of a plethora of target genes involved in multiple cellular events of tumorigenesis, including inflammation, proliferation, survival, angiogenesis, invasion and metastasis [69,70,71]. Empirical evidence suggests a critical link between NF-κB-mediated inflammation and breast cancer development and progression [29,36]. NF-κB undergoes persistent (constitutive) activation in a variety of breast cancers, including IBC [35,72,73]. In patients with invasive ductal carcinoma, NF-κB p65 overexpression has been associated with advanced stage, large tumor size, high grade and high Nottingham prognostic index [74]. Suppression of the constitutive activation of NF-κB diminishes the oncogenic potential of transformed cells and inhibition of this proinflammatory pathway may provide novel opportunities for prevention as well as treatment of cancer [75,76,77]. In our present study, low expression of NF-κB p65 as well as IκBα in cytosol and high expression of NF-κB p65 in nucleus of mammary tumor cells from DMBA control animals suggest possible degradation of IκBα, subsequent release of activated NF-κB p65 and its eventual translocation to the nucleus. These results corroborate with an earlier study which showed that activation of NF-κB plays an early, critical role in DMBA-initiated malignant transformation in rat mammary glands [78]. Other laboratories also reported high levels of NF-κB binding activities and elevated protein levels of NF-κB p65 in DMBA-induced mammary tumors in rodents [79,80]. Our findings of TPE-mediated protection against IκBα degradation and interference with translocation of activated NF-κB p65 to the nucleus indicate that TPE possibly abrogates early critical events implicated in DMBA-induced mammary tumorigenesis in rats. Consistent with our results, several investigators reported a suppression of NF-κB in conjunction with chemopreventive efficacy of several natural agents against DMBA-induced rat mammary tumorigenesis [54,56,64,80].

The transcriptional factor Nrf2 is known to play a decisive role in protecting mammalian cells against inflammation as well as oxidative and electrophilic stresses [81]. A growing body of evidence indicates that the Nrf2 signaling pathway is intimately involved with the regulation of inflammation as well as inflammation-associated carcinogenesis and hence this pathway represents an important target for chemoprevention of inflammation-linked carcinogenesis [82,83,84,85,86]. Interestingly, the Nrf2 signaling pathway is implicated in the suppression of NF-κB-mediated inflammatory effects [87] and conversely, ablation of Nrf2 seems to accelerate proinflammatory reactions facilitated by NF-κB [88,89]. Emerging evidence underscores a potential crosstalk between Nrf2 and NF-κB transcription factors modulated through the mitogen-activated protein kinase cascade that may influence the inflammation-associated etiopathogenesis of cancer [38,39]. Using Nrf2 knockout (KO) mice, Becks and colleagues [90] investigated the role of Nrf2 in mammary gland carcinogenesis induced by DMBA. Compared to the wild-type controls, the KO mice had significantly lower mammary tumor-free survival and exhibited rapid, aggressive mammary carcinoma progression with concomitant increase (2-fold) in NF-κB activation in mammary carcinomas. Decreased protein and mRNA expression of Nrf2 and Nrf2-regulated genes were observed in estrogen-exposed mammary tissue and mammary tumors in rats [91,92,93]. Yao *et al*. [94] reported inhibitory effects of the estrogen receptor signaling pathway on Nrf2-dependent enzymes in breast cancer. Consistent with the aforementioned studies, we have also observed suppression of Nrf2 in rat mammary tumors induced by DMBA. Moreover, the ability of TPE to upregulate the expression of intratumor Nrf2 and its nuclear translocation may lead to the induction of various antioxidant and detoxifying enzymes to limit DMBA-induced oxidative stress which is known to activate NF-κB. It is also likely that Nrf2 may directly suppress NF-κB. Thus, TPE may directly or indirectly suppress the NF-κB-mediated inflammatory response and ultimately contribute to breast cancer prevention. Our findings are supported by a recent study showing the involvement of Nrf2 transactivation in dietary extra-virgin olive oil-mediated chemoprevention of DMBA-induced mammary carcinogenesis in rats [95]. Chen and colleagues [96] also showed curcumin-mediated inhibition of breast cancer cell proliferation through upregulation of Nrf2 protein. Since there have been numerous reports that the activation of Nrf2 and its target genes can favor tumor growth [97], additional studies are warranted to understand the full implication of TPE-induced Nrf2 expression during mammary tumorigenesis.

The exact bioactive phytochemicals of TPE responsible for the observed anti-inflammatory activities during breast carcinogenesis are not known at the present time and require additional comprehensive studies. Phytochemical screening of *T. portulacastrum* reveals the presence of several constituents, including ecdysterone, trianthenol, 3-acetylaleuritolic acid, 5,2'-dihydroxy-7-methoxy-6,8-dimethylflavone, leptorumol, 3,4-dimethoxy cinnamic acid, 5-hydroxy-2-methoxybenzaldehyde, *p*-methoxybenzoic acid and beta cyanin [11]. In view of the mounting evidence that plant phytochemicals exhibit cancer preventive and anticancer effects when they are used in combination rather than individually [98,99,100], it is tempting to speculate that various bioactive phytoconstituents of *T. portulacastrum* may regulate pro-inflammatory molecules and pathways during mammary carcinogenesis through a synergistic effect.

Based on the results presented here, we conclude that TPE inhibits the inflammatory cascade during DMBA-induced rat mammary gland carcinogenesis by modulating several inflammatory and stress mediators, namely COX-2, HSP90, NF-κB, and Nrf2. These results provide mechanistic insight of our previously reported findings that TPE exhibits chemopreventive effects on DMBA-induced mammary tumorigenesis in rats through its antiproliferative and proapoptotic activities [27]. TPE may suppresses DMBA mammary carcinogenesis by blocking degradation of IκBα, impeding activation and subsequent translocation of NF-κB from cytosol to nucleus, hampering DNA-NF-κB binding and disrupting the trans-activation of NF-κB-regulated genes. The NF-κB-driven genes affected by TPE during rat mammary carcinogenesis include COX-2 as showed in the present study as well as Bcl-2 and cyclin D1 as reported in our earlier communication [27]. TPE-mediated suppression of NF-κB signaling could, at least in part, be achieved by activation of its negative regulator—the Nrf2 pathway. Our present findings in conjunction with our previous results suggest that TPE prevents DMBA-induced breast neoplasia by anti-inflammatory mechanisms mediated through simultaneous and differential modulation of two interconnected molecular circuits, namely the NF-κB and Nrf2 signaling pathways. The precise mechanisms by which TPE inhibits NF-κB and activates Nrf2 are not clear at the present time and we would like to conduct additional experiments in the future to better understand TPE action on NF-κB and Nrf2. Since animal models do not always translate to human situations, additional clinical studies are also warranted to understand the full translational impact of *T. portulacastrum* for human breast cancer prevention and intervention.

## 4. Experimental Section

### 4.1. Plant Material

The ethanolic extract of aerial parts of *T. portulacastrum* (TPE) was prepared following our previously published procedure [12] and used in this study. We previously reported the results on high performance liquid chromatography-fingerprint analysis of this extract [12].

### 4.2. Chemicals and Antibodies

The chemical carcinogen DMBA was purchased from Sigma-Aldrich (St. Louis, MO, USA). Paraformaldehyde was obtained from Ted Pella (Redding, CA, USA). Primary antibodies, such as COX-2, NF-κB p65, IκBα, Nrf2 as well as ABC staining kit were procured from Santa Cruz Biotechnology (Santa Cruz, CA, USA). HSP90 was obtained from Enzo Life Sciences (Farmingale, NY, USA).

### 4.3. Experimental Design and Tissue Harvesting

Breast tumor samples utilized in the current investigation were harvested from our previously reported chemopreventive study in which female Sprague-Dawley rats (Harlan Laboratories, Indianapolis, IN, USA) subjected to dietary administration of TPE at 50, 100 and 200 mg/kg body weight for 18 consecutive weeks exhibited 11%, 22% and 42% inhibition of the incidence of DMBA-induced mammary tumors, respectively [27]. The animal experimentation was conducted following an animal protocol approved by the Northeast Ohio Medical University Institutional Animal Care and Use Committee (Rootstown, OH, USA). In short, rats (at approximately 36 days of age) were randomly distributed in four groups of 5–11 animals each. One animal group was maintained on a basal rodent diet (LabDiet, St. Louis, MO, USA) without any further dietary treatment, whereas the remaining three groups had access to the same basal diet supplemented with TPE to yield three dietary doses of the extract *i.e.*, 50, 100 and 200 mg/kg body weight. Following 2 weeks of the aforementioned treatment protocol, mammary tumorigenesis was initiated in all animals by a single oral administration of DMBA (50 mg/kg body weight) as per our previous report [101]. Feeding of rats with TPE was continued for another 16 weeks following the DMBA treatment. The animal experimentation was terminated at 16 weeks post-DMBA treatment (*i.e*., 18 weeks following the initiation of the experiment). Mammary tumors were excised from various groups under anesthesia and tumor samples were fixed in 4% paraformaldehyde. Serial sections (approximately 15 μm thick) were cut using a microtome under freezing condition, kept in a −80 °C freezer and subsequently used for immunohistochemical assays.

### 4.4. Immunohistochemical Analysis

The protein expressions of COX-2, HSP90, NF-κB p65, IκBα and Nrf2 in mammary tumor sections were determined by methods we described previously [102]. Briefly, the tissue sections were first hydrated using 1× phosphate-buffered saline (PBS) for 5 min followed by incubation in sodium citrate buffer (10 mM, pH 6.0) for 10 min at 80 °C for antigen retrieval. All subsequent steps were performed at room temperature. Endogenous peroxidases were blocked by treating the samples with 1% H_2_O_2_ in PBS for 5 min followed by washing with PBS for 5 min. Tissue sections were then treated with blocking solution for 1 h followed by washing with PBS and incubating overnight (at 4 °C) with primary antibodies (COX-2, HSP90, NF-κB, IκBα or Nrf2) at 1:100 dilution. Following several washes, tissue sections were treated with horseradish peroxidase-conjugated secondary antibody (1:200 dilution) for 30 min at room temperature and then with 3,3'-diaminobenzidine tetrahydrocholoride solution to visualize brown antigen-antibody complexes. Finally, sections were counterstained with Gill’s hematoxylin solution, air dried and mounted using DPX (Electron Microscopy Sciences, Hatfield, PA, USA). The immunohistochemical slides were visualized under a light microscope (BX43, Olympus, Center Valley, PA, USA) and 1000 tumor cells/animal were analyzed. All immunohistochemical results were expressed as percentage of immunopositive cells.

### 4.5. Statistical Analyses

All results are expressed as mean ± SEM. Statistical analyses were performed by using commercial software SigmaPlot 11.0 (Systat Software, Inc., San Jose, CA, USA). One-way analysis of variance with least significant difference *post hoc* analysis was also employed to compare various parameters among different treatment and control groups. A *p* value less than 0.05 was considered significant.
